# Cancer survival discrepancies in developed and developing countries: comparisons between the Philippines and the United States

**DOI:** 10.1038/sj.bjc.6604945

**Published:** 2009-02-24

**Authors:** M T Redaniel, A Laudico, M R Mirasol-Lumague, A Gondos, D Pulte, C Mapua, H Brenner

**Affiliations:** 1 Division of Clinical Epidemiology and Aging Research, German Cancer Research Center, Bergheimer Str 20, D-69115, Heidelberg, Germany; 2 Manila Cancer Registry, Philippine Cancer Society, 310 San Rafael St, San Miguel, 1005 Manila, Philippines; 3 Department of Surgery, Philippine General Hospital, University of the Philippines-Manila, Taft Avenue, 1000 Manila, Philippines; 4 Department of Health-Rizal Cancer Registry, Rizal Medical Center, Pasig Boulevard, 1600 Pasig City, Philippines; 5 Department of Medicine, Division of Hematology/Oncology, University of Medicine and Dentistry of New Jersey, Newark, NJ, 07101, USA; 6 Bioinformatics Department, Research and Biotechnology Division, St. Luke's Medical Center, 279 E. Rodriguez Sr. Boulevard, 1112 Quezon City, Philippines

**Keywords:** cancer survival, developing countries, Philippines, Filipino-Americans, period analysis

## Abstract

Despite the availability of population-based cancer survival data from the developed and developing countries, comparisons remain very few. Such comparisons are important to assess the magnitude of survival discrepancies and to disentangle the impact of ethnic background and health care access on cancer survival. Using the SEER 13 database and databases from the Manila and Rizal Cancer Registries in the Philippines, a 5-year relative survival for 9 common cancers in 1998–2002 of Filipino-American cancer patients were compared with both cancer patients from the Philippines, having the same ethnicity, and Caucasians in the United States, being exposed to a similar societal environment and the same health care system. Survival estimates were much higher for the Filipino-Americans than the Philippine resident population, with particularly large differences (more than 20–30% units) for cancers with good prognosis if diagnosed and treated early (colorectal, breast and cervix), or those with expensive treatment regimens (leukaemias). Filipino-Americans and Caucasians showed very similar survival for all cancer sites except stomach cancer (30.7 *vs* 23.2%) and leukaemias (37.8 *vs* 48.4%). The very large differences in the survival estimates of Filipino-Americans and the Philippine resident population highlight the importance of the access to and utilisation of diagnostic and therapeutic facilities in developing countries. Survival differences in stomach cancer and leukaemia between Filipino-Americans and Caucasians in the United States most likely reflect biological factors rather than the differences in access to health care.

Despite the availability of population-based cancer survival data from the developed and developing countries (Sankaranarayanan *et al*, 1998; Verdecchia *et al*, 2007), comparisons between developing and developed countries remain very few (Gondos *et al*, 2004, 2005; Coleman *et al*, 2008). Comparison of cancer survival between countries or between ethnic population groups within the same country may yield important information regarding the magnitude of discrepancies in access to and utilisation of health care between population groups. Furthermore, they could also give insights on possible ethnic differences in tumour biology.

Moreover, the survival experiences of similar ethnic populations in developed and developing country settings have rarely been compared, mostly because of the absence of data from the developing countries. Very few population-based studies have been conducted focusing on cancer survival in the Philippines and of those that exists; most are outdated (Sankaranarayanan *et al*, 1998). Nevertheless, it has been shown that the survival estimates of cancer patients in the Philippines were lower than those found in developed countries (Sankaranarayanan *et al*, 1998). No earlier study has included comparisons between the survival experience of Filipinos in the Philippines and Filipinos residing in developed countries. As various studies have suggested that access to health care is a key factor affecting survival, the differences between settings should be investigated.

The aim of this study was to assess the magnitude of survival discrepancies and to disentangle the impact of ethnic background and access to health care by comparing cancer survival in Filipino-Americans to cancer patients from the Philippines, having the same ethnicity, and to Caucasians in the United States, being exposed to a similar societal environment and the same health care system.

## Materials and methods

For Filipino-Americans, data from the SEER 13 database (Surveillance Epidemiology and End Results (SEER) Program) for patients identified to be of Filipino origin, regardless of birthplace, and diagnosed with the cancers of interest at the age of 15 years or above from 1 January 1993 to 31 December 2002 were selected. Cancer patients who were identified by death certificates only (DCO) and *in situ* cases were excluded. Data for a total of 13 204 patients were used for the survival analysis.

For Caucasians, using the same inclusion and exclusion criteria, 612 244 patients diagnosed with the cancers of interest between 1993 and 2002 whose race or ethnicities were coded as white, including those coded as being of hispanic ethnicity, were included in the analysis.

For cancer patients from the Philippines, data from the Philippine Cancer Society-Manila Cancer Registry (PCS-MCR) and the Department of Health-Rizal Cancer Registry (DOH-RCR) were used. These registries cover the national capital region (NCR), more commonly known as Metro Manila, with a population of 9 932 560 (2000 census; NSCB, 2006).

For this survival study, subsamples of cancer patients who were diagnosed at the age of 15 years or above between 1993 and 2002 were included in the analysis. From the lists of colorectal, breast, ovarian and cervical cancer patients who met the inclusion criteria, samples of 200 cases per site for each year studied were randomly selected. For leukaemias, stomach, liver, lung and thyroid cancers, samples of 100 patients per year per site were randomly selected. Survival status was assessed through death certificate notifications mentioning cancer as the cause of death, which were collected from the Local Civil Registry Offices and matched with records from the registry database. For those not identified as dead, active follow-up by personal visits to the patients or their families in the last known place of residence was used to confirm status. Of those not matched to death certificates, complete 5-year survival was obtained for 41.6% of the cases, whereas some survival information was obtained for an additional 39%.

The original sample included 13 000 patients. After all exclusions because of invalid data (*n*=1102, 8.5%) and lack of any follow-up data (*n*=1734, 13.3%), 10 164 patients (78.2%) were left for the survival analysis. Anonymised data sets were prepared and used in the analysis.

The project proposal was approved by the Ethics Review Board of the National Institutes of Health of the University of the Philippines, Manila. The information obtained strictly conformed to the code of conduct stipulated by the guidelines on confidentiality for population-based cancer registries (International Association of Cancer Registries and International Agency for Research on Cancer, 2004). Informed consent was obtained from patients who were followed-up at the places of residence.

### Data analysis

Traditionally, cohort-based analysis, which includes the conventional life table (actuarial) method or the Kaplan–Meier method (Cutler and Ederer, 1958; Kaplan and Meier, 1958) has mostly been used to derive survival estimates. Here, period analysis, a new method of survival analysis introduced by Brenner and Gefeller in 1996 was used (Brenner and Gefeller, 1996). It has been shown that period analysis provides more up-to-date estimates of survival that closely predicts survival later observed for patients diagnosed in the respective period (Brenner *et al*, 2002; Brenner and Hakulinen, 2002a, 2002b; Tälback *et al*, 2004; Ellison, 2006).

According to common practice in population-based cancer survival analysis, relative rather than absolute survival is reported. Relative survival reflects the survival experience of cancer patients in the absence of competing causes of death (Ederer *et al*, 1961; Henson and Ries, 1995). It is calculated as the ratio of the observed survival of the cancer patients and the expected survival of a group of people with the same age and sex distribution from the general population. For all groups, expected survival was derived from life tables for the year 2000 using the so-called Ederer II method (Ederer and Heise, 1959). The life table for the US population was obtained from the US National Center for Health Statistics (Arias, 2002). Because of the absence of life tables for other races for 2000, the life table for whites was used for both SEER populations. The life table for the Philippine resident population was derived from the projected population estimate and the actual mortality data for this area, which were obtained from the Philippine National Statistics Office (NSO).

To compare the survival estimates between the different cancer populations, age adjustment is warranted. For this purpose, age-specific period survival estimates for age groups 15–44, 45–54, 55–64, 65–74 and 75 years and above were obtained from the Philippine resident, the Filipino-American and from the Caucasian populations. For each of the cancer populations, the age-specific estimates were weighted and then summed. Weights used were taken from the World Standard Cancer Patient Population (WSCPP; Sankaranarayanan *et al*, 1998), with the exception of thyroid cancer. In the absence of an appropriate standard population for thyroid cancer in WSCPP, the International Cancer Survival Standards suggested by Corazziari *et al* (2004) were used.

The differences between relative survival estimates for the different cancer populations were tested for statistical significance using a novel modelling approach for period analysis (Brenner and Hakulinen, 2006). First, age-specific numbers of patients at risk and of deaths by year of follow-up were calculated separately for each group. Then, Poisson regression models were fitted, in which the numbers of deaths were modelled as a function of the population group (Philippine residents or Filipino-Americans or Caucasians), year of follow-up (1, 2, 3, 4, 5 – entered as a categorical variable) and age-group (15–44, 45–54, 55–64, 65–74, 75+ – entered as a categorical variable), using the logarithm of the person-years at risk as offset, and accounting for late entries and withdrawals as half persons. This approach allowed testing for significance of differences in relative survival after adjustment for age, based on *P*-values for the population parameter estimate. A significance level of *α*=0.05 (two-sided testing) was employed.

All analyses were performed with the SAS Statistical Analysis Software, using special macros for standard and modelled period survival analysis as described previously (Brenner *et al*, 2004b; Brenner and Hakulinen, 2006).

## Results

Table 1 shows the numbers of cases, the mean age at diagnosis and the proportions of female and histologically verified cases for the Philippine resident, Filipino-American and Caucasian patients. For most forms of cancer, the US Caucasian patients were on average about 10 or more years older than Philippine resident patients, with Filipino-American patients having intermediate mean ages. The age difference was most pronounced for leukaemia. In contrast, mean ages were rather similar across groups for patients with cervical and thyroid cancers. For most cancers, the proportions of male and female cases were similar between the three groups, with the exception of stomach cancer where the proportion of female cases was higher in the Philippines, and in liver and lung cancers where the proportion of female cases was higher among Caucasian patients. In all three populations, the vast majority of cancers were histologically verified. However, the proportion of histologically verified cases was markedly lower in the Philippines than in the other populations for cancers of the stomach, liver and lung.

The age-adjusted 5-year relative survival estimates for the 1998–2002 period are shown in Table 2. In all three populations, patients with thyroid cancer had the highest 5-year relative survival (82.4, 91.3 and 92.3% for the Philippine resident, Filipino-American and Caucasian patients, respectively). Liver cancer showed the lowest survival among the Filipino-Americans (11.7%) and Caucasian patients (12.3%), whereas survival was lowest for patients with leukaemia (5.2%) in the Philippines.

Comparing Filipino-American and the Philippine resident patients, all survival estimates for Filipino-American patients were higher. The differences in survival estimates ranged from 3.2% units for liver cancer to 32.6% units for leukaemias. With the exception of stomach and liver cancer, all of the differences were statistically significant.

Between Filipino-Americans and Caucasian patients, survival differences were mostly small and statistically not significant. However, survival was substantially and significantly higher among Filipino-American than among Caucasian patients with stomach cancer (30.7 *vs* 23.2%, *P*=0.04), whereas the opposite was true for patients with leukaemia (37.8 *vs* 48.4%, *P*<0.0001). Comparing the Filipino-American and Caucasian stomach cancer patients, more Filipino-Americans were diagnosed with non-cardia gastric cancer (82.2 *vs* 63.2%). For leukaemias, more Filipino-Americans were diagnosed with acute myeloid leukaemia (AML; 33.2 *vs* 19.4%), whereas more Caucasians were diagnosed with chronic lymphocytic leukaemia (CLL; 40.2 *vs* 9.8%).

## Discussion

This study shows one of the few comparisons of cancer survival between developed and developing country settings. To our knowledge, this is the first comprehensive comparison of cancer survival of Filipino-American cancer patients with both cancer patients from the Philippines, who have the same ethnicity, and Caucasians in the United States, who share a similar societal environment and the same health care system. Through the application of period analysis, up-to-date survival estimates could be obtained.

When compared with the Filipino-American patients, the survival estimates for the Philippine resident patients are generally much lower. Survival estimates are similar only for cancer sites for which the chances of cure are low even in developed countries (liver, lung and stomach cancers). By contrast, large differences are seen for cancers associated with a moderate-to-good prognosis if diagnosed and adequately treated early (colorectal, breast and cervical cancers), suggesting that the stage at diagnosis and quality of treatment are important determinants of survival. On the basis of the registry data, among the Filipino-American breast cancer patients with staging information, 55.8% were diagnosed to have tumours at T0-1 stages, whereas this proportion was only 11.8% in the Philippine resident population. For cervical cancer, 55.6 and 23.9% of cases were identified to have stage I (International Federation of Gynecology and Obstetrics (FIGO)) cancers among Filipino-American and Philippine resident patients, respectively.

Survival estimates are also strongly divergent for cancers for which effective but very expensive treatment regimens are available such as leukaemias. This pattern reflects low access of the Philippine population to such therapeutic regimens. However, for ovarian cancer, in spite of the high cost of therapy, survival for the Filipino-Americans was only moderately higher than for patients in the Philippines. One possible explanation could be that the patients in the Philippines were less often diagnosed with clear cell carcinoma (6.1 *vs* 10.2%), which is associated with poorer prognosis (Chan *et al*, 2008).

The differences between the Filipino resident population and Filipino-Americans suggest continued inadequacy of access to or utilisation of diagnostic and therapeutic procedures in the Philippines. Although diagnostic and treatment facilities are available, access for a majority of cancer patients is still a problem. For most part, these services are costly for the average Filipino as these are mainly offered by private hospitals and clinics. Government facilities offer subsidised services, but these are limited, with public hospitals accounting for only 25% of the total number of hospitals in the NCR (NSCB, 2006). Moreover, most Filipino cancer patients seek medical advice only when symptomatic or in the advanced stages (Ngelangel and Wang, 2001). In spite of the high level of education of the population in the NCR, there seems to persist a lack of trust in one's chances to be cured (Pisani *et al*, 2006). Regardless of health information campaigns, a diagnosis of cancer is still viewed by many as life-threatening.

Between the Filipino-American and Caucasian patients, survival is comparable for most cancers. These results are consistent with those of earlier studies mostly focusing on single forms of cancers (Flood *et al*, 2000; Pineda *et al*, 2001; Lin *et al*, 2002; McGuire *et al*, 2002; Hashibe *et al*, 2003; Pagano *et al*, 2003; Chien *et al*, 2005; Gomez *et al*, 2007; Sun *et al*, 2007). Existing differences probably lie more in biological factors than health care-related factors. The difference in the survival estimates for stomach cancer could be explained by the higher proportion of non-cardia gastric cancer cases in Filipino-American patients than in Caucasian patients. Non-cardia gastric cancer is strongly associated with *Helicobacter pylori* infection (Brenner *et al*, 2004a) and higher relapse-free and overall survival (Verdecchia *et al*, 2004). For leukaemias, the survival advantage of Caucasian patients may reflect the higher proportion of CLL, which is associated with good survival (Xie *et al*, 2003; Brenner *et al*, 2008) and the lower proportion of AML, which is associated with poor survival (Xie *et al*, 2003; Pulte *et al*, 2008). The survival estimates of Filipino-Americans were higher than those of Caucasians for ovarian cancer, although the difference was not statistically significant. Although more Filipino-Americans were diagnosed with clear cell carcinomas (10.0 *vs* 4.9%), Filipino-Americans were younger, and a higher proportion was diagnosed at an earlier stage (FIGO stage I, 36.0 *vs* 24.5%).

For the Filipino-American population, one factor that could have an effect on survival is acculturation. It can be argued that the degree of acculturation of immigrants might have an impact on survival, with those more acculturated having better access to and greater utilisation of health care facilities, particularly those concerning the early detection, diagnosis and quality treatment of certain types of cancer. In two earlier studies, foreign-born Asian-Americans had lower survival than those born in the United States (Choe *et al*, 2005; Chuang *et al*, 2006). However, acculturation did not have an effect on cancer survival in a study focusing on Asian-American patient groups, including Filipino-Americans (Pineda *et al*, 2001). In our study population, almost 70% of the Filipino-American patients were born in the Philippines, and the comparably high survival rates of this ethnic group, compared with the Caucasian residents, suggest a rapid and profound acculturation.

Some limitations should be considered in the interpretation of these survival estimates. Population-based survival figures are influenced by a variety of factors, including those related to cancer services, such as organisation, training and skills of health care professionals, application of guidelines and diagnostic and treatment facilities, and clinical factors, such as tumour stage and biology (Verdecchia *et al*, 2007). With the use of age standardisation and the relative survival methodology, the impact of age differences should be minimised. Unfortunately, information on stage distribution was incomplete and not fully comparable for most cancers in both Philippine registries. The same applies to the information on various types of therapy and socioeconomic indices, which limits the possibilities to explore the issues underlying the survival differences in more detail.

The differences in cancer survival between Filipino-American and Philippine resident patients confirm the importance of access to diagnostic and therapeutic facilities.

## Figures and Tables

**Table 1:** Description of cancer patients included in the period survival analysis, Philippine residents and Filipino-Americans and Caucasians from the US SEER, 1993–2002

	**Philippine residents**				**Filipino-Americans, the US SEER**				**Caucasians, the US SEER**			
**Site**	***N***	**Mean age (±s.d.)**	**% Female cases**	**% HV^a^**	***N***	**Mean age (±s.d.)**	**% Female cases**	**% HV^a^**	***N***	**Mean age (±s.d.)**	**% Female cases**	**% HV^a^**
Stomach	792	60.2 (14.2)	46.0	82.1	516	67.8 (13.9)	39.2	99.0	19432	69.3 (13.9)	37.8	98.5
Colorectal	1635	58.8 (14.7)	48.0	92.0	2598	65.6 (13.9)	43.3	98.8	139479	70.4 (12.9)	49.5	98.7
Liver	772	56.4 (14.8)	25.1	49.2	570	64.2 (14.2)	25.4	78.4	10078	66.6 (13.3)	33.6	78.8
Lung	840	59.9 (11.7)	26.9	79.6	2932	67.7 (11.4)	29.8	95.6	156286	68.7 (10.9)	45.3	93.6
Leukaemia	718	43.5 (19.9)	49.7	98.5	483	59.3 (18.8)	44.3	98.1	29812	64.9 (17.4)	41.8	97.0
Breast	1615	51.4 (12.7)	100.0	95.0	4203	55.5 (12.4)	100.0	99.9	202928	62.4 (14.4)	100.0	99.4
Cervix	1580	49.8 (12.3)	100.0	98.1	479	53.0 (13.9)	100.0	99.6	13285	49.2 (15.8)	100.0	99.6
Ovary	1475	48.2 (15.2)	100.0	90.5	448	54.1 (14.6)	100.0	98.0	21052	62.5 (15.4)	100.0	96.5
Thyroid	737	45.8 (15.9)	81.8	96.1	975	49.1 (16.0)	79.4	99.9	19892	47.4 (16.3)	74.7	99.9

s.d.=standard deviation.

a Histologically verified cases.

**Table 2:** A 5-year relative survival (%) of cancer patients adjusted to the World Standard Cancer Patient Population, Philippine residents and Filipino-Americans and whites from the US SEER, 1998–2002

	**(1) Philippine residents**		**Between (1) and (2)**		**(2) Filipino-Americans**		**Between (2) and (3)**		**(3) Caucasians**	
**Site**	**Period estimate**	**s.e.**	**Difference**	***P*-value**	**Period estimate**	**s.e.**	**Difference**	***P*-value**	**Period estimate**	**s.e.**
Stomach	27.3	4.9	3.4	0.09	30.7	3.6	−7.5	0.04	23.2	0.5
Colorectal	40.2	4.4	22.0	<0.0001	62.3	1.9	1.8	0.72	64.0	0.2
Liver	8.5	1.9	3.2	0.06	11.7	2.4	0.6	0.89	12.3	0.7
Lung	12.0	3.7	6.0	<0.0001	17.9	1.2	−0.7	0.06	17.3	0.2
Leukaemia	5.2	1.5	32.6	<0.0001	37.8	3.8	10.6	<0.0001	48.4	0.5
Breast	58.6	4.1	31.0	<0.0001	89.6	1.2	−1.3	0.51	88.3	0.1
Cervix	45.4	3.7	21.7	<0.0001	67.2	3.3	0.2	0.68	67.4	0.7
Ovary	49.5	4.8	6.6	0.03	56.1	3.4	−5.9	0.35	50.2	0.5
Thyroid^a^	82.4	5.7	8.9	0.00003	91.3	2.4	1.0	0.71	92.3	0.5

s.d.=standard error.

a Adjusted to the International Cancer Survival Standards suggested by Corazziari *et al* (2004).
